# Association between physical activity domains and cardiovascular diseases among US adults: evidence from NHANES 2007–2020

**DOI:** 10.1038/s41598-025-19419-3

**Published:** 2025-10-09

**Authors:** Bingwen Huang, Canye Lin, Yuhuan Feng, Shanshan Cai, Mujuan Zhao, Chaochao Hu, Linlin Wang

**Affiliations:** 1 Department of Sports Science, College of Sports & Arts, Hanyang University ERICA Campus, Seoul, South Korea; 2 College of Sports and Health Sciences, Guizhou Medical University, Guiyang, Guizhou China; 3 Department of Biomedical Engineering, The Hong Kong Polytechnic University, Hongkong, China; 4 College of Sports and Health Sciences, Medicine & Technology College of Zunyi Medical University, Zunyi, Guizhou China; 5 Division of Biomedical and Life Sciences, Faculty of Health and Medicine, Lancaster University, Lancaster, UK; 6 Department of sports sociology, Kyungpook National University, Sangju, South Korea; 7 Martial Arts Academy, Guangzhou Sport University, Guangzhou, Guangdong China

**Keywords:** Cardiovascular disease, Physical activity, Leisure-time physical activity, Occupational physical activity, Transportation-related physical activity, Risk factors, Occupational health, Epidemiology

## Abstract

This study examined the associations between different domains of physical activity, leisure-time (LTPA), occupational (OPA), and transportation-related (TPA), and cardiovascular disease (CVD) risk among U.S. adults. Data from the National Health and Nutrition Examination Survey (NHANES) 2007–2008 through March 2020 were used. Physical activity was self-reported using the Global Physical Activity Questionnaire. Weekly minutes of LTPA, OPA, and TPA were calculated and categorized as: <150, 150–299, and ≥ 300 min. CVD was defined as self- or proxy-reported heart failure, coronary heart disease, angina, heart attack, or stroke. Weighted multivariable logistic regression was used to assess associations. Six sensitivity analyses were conducted. Meeting the physical activity guidelines (≥ 150 min/week) through LTPA or TPA was associated with significantly lower odds of CVD, 22% [OR: 0.78, 95% CI: 0.70–0.88] and 40% [OR: 0.60, 95% CI: 0.50–0.72], respectively. This was supported by sensitivity analyses. In a separate dose-response analysis, individuals engaging in twice the recommended minimum (≥ 300 min/week) experienced even greater reductions in CVD risk, with 30% lower odds for LTPA [OR: 0.70, 95% CI: 0.60–0.80] and 41% lower odds for TPA [OR: 0.59, 95% CI: 0.47–0.74]. In contrast, OPA was not significantly associated with CVD risk at any activity level. Among U.S. adults, higher LTPA and TPA are associated with lower CVD risk, particularly at higher levels of activity. In contrast, OPA does not show the same protective effect. The associations between physical activity and CVD risk vary by context.

**Supplementary Information:**

The online version contains supplementary material available at 10.1038/s41598-025-19419-3.

## Introduction

Cardiovascular disease (CVD) remains one of the leading causes of illness and death worldwide, placing a substantial burden on global health systems and economies^1^. Over the past few decades, the number of people living with CVD has nearly doubled, from 271 million in 1990 to 523 million in 2019 ^2^. Despite progress in diagnosis and treatment, CVD-related deaths have continued to rise, increasing from 12.1 million in 1990 to 18.6 million in 2019^2^. This growing trend is largely driven by population aging and ongoing exposure to major risk factors^2^. Among the modifiable lifestyle factors, physical activity stands out as a key element in preventing and managing CVD^3^. Regular physical activity is not only linked to a lower risk of developing CVD but also helps slow disease progression and improve long-term outcomes for those already living with the condition^3,4^.

While the protective effects of physical activity on cardiovascular health are well established, not all forms of physical activity appear to offer the same benefits. Physical activity occurs across various domains, including leisure-time physical activity (LTPA), occupational physical activity (OPA), and transportation-related physical activity (TPA)^5^. LTPA is typically voluntary and includes recreational sports or structured exercise, often accompanied by sufficient rest and recovery^6^. In contrast, OPA consists of physical tasks performed as part of one’s job, which are often repetitive, performed under time constraints, and may involve inadequate recovery^7,8^. TPA primarily includes walking or cycling as a means of commuting^9^. Emerging evidence suggests that the health impacts of physical activity may vary depending on the domain^5,10–12^.

Although prior research has explored the associations between OPA and LTPA and CVD, existing studies often face methodological limitations. For example, in a U.S. study, OPA was assessed using Likert-scale responses on exertion and standing/walking at work^8^, an approach that does not quantify activity volume and limits comparability with guideline-based recommendations, which are typically time-based. TPA, a distinct and increasingly relevant domain, has seldom been evaluated concurrently with other physical activity domains in the same population^8,13^. Moreover, some studies relied on a narrow range of survey years^8,13^, limiting temporal scope and generalizability. These limitations highlight the need for further research using time-based metrics across all activity domains and broader data coverage to better understand how different types of physical activity relate to CVD risk.

Therefore, this study aimed to investigate the associations between different physical activity domains (LTPA, OPA, and TPA) and the risk of CVD in U.S. adults using the National Health and Nutrition Examination Survey (NHANES), 2007–2020. Specifically, we first assessed whether meeting the current physical activity guidelines was associated with CVD risk, and then explored potential dose-response relationships across different activity levels. Given that sociodemographic factors such as age, sex, educational attainment, poverty-income ratio (PIR), and marital status may influence both physical activity behaviors and CVD risk, we also evaluated whether the associations between physical activity and CVD risk differed across these characteristics. By differentiating among physical activity domains and investigating subgroup differences, we sought to provide more comprehensive insights into how specific patterns of physical activity influence cardiovascular health, ultimately informing more targeted and effective public health strategies.

## Study population

This cross-sectional study utilized data from the NHANES (https://www.cdc.gov/nchs/nhanes/index.html). NHANES is a nationally representative survey designed to assess the health and nutritional status of the U.S. population. It employs a complex, multistage probability sampling method to select participants from the non-institutionalized civilian population across the United States. Data collection involves structured interviews, physical examinations, and laboratory tests. The survey protocol was approved by the National Center for Health Statistics Ethics Review Committee, and all data is publicly available. Therefore, no additional ethical approval was required for this secondary analysis.

**Fig. 1:**
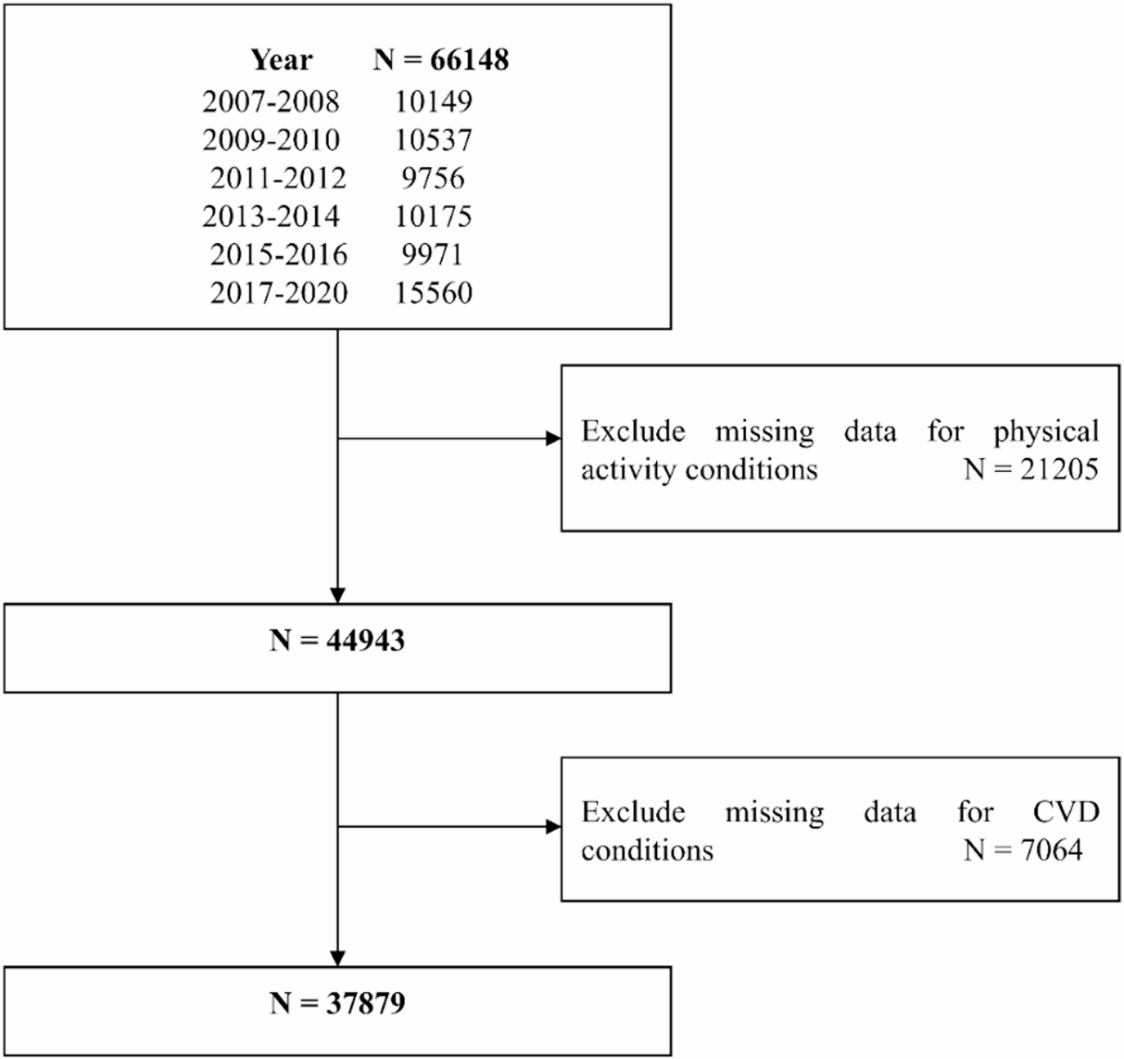
Flowchart of selecting data from the NHANES 2007-March, 2020. CVD, Cardiovascular disease.

To minimize the potential impact of altered physical activity patterns due to remote work during the COVID-19 pandemic, we restricted our analysis to data collected before the pandemic, specifically, from the 2007–2008 cycle through March 2020. After excluding individuals with missing data on key exposure variables (physical activity measures) or outcome variables (CVD conditions), a total of 37,879 participants aged 20 years and older were included in the final analysis (Fig. 1).

### Physical activity

Physical activity levels were assessed using the Global Physical Activity Questionnaire (GPAQ), a validated instrument developed by the World Health Organization (WHO). This questionnaire captures information on the frequency (days per week), duration (minutes per day), and intensity (moderate or vigorous) of physical activity across three distinct domains: OPA, TPA, and LTPA. To estimate total energy expenditure, vigorous-intensity physical activity was converted into an equivalent amount of moderate-intensity activity, with one minute of vigorous activity counted as two minutes of moderate activity, an approach supported by previous research^16^. Using this conversion, we calculated the total weekly minutes of OPA and LTPA by doubling the time spent in vigorous activity and adding it to the time spent in moderate activity. TPA, which primarily included walking and cycling, was classified as moderate in intensity.

In line with the 2020 WHO physical activity guidelines, adults, including those with chronic conditions, are advised to engage in 150 to 300 min of moderate-intensity activity, 75 to 150 min of vigorous-intensity activity, or an equivalent combination each week^17^. Based on these recommendations, participants were initially categorized into two groups: (1) those who met the minimum recommended activity level (≥ 150 min/week of moderate-intensity equivalent activity), and (2) those who did not (< 150 min/week). For further analysis, we classified participants into three groups based on total weekly activity: (1) less than 150 min, (2) 150 to 299 min, and (3) 300 min or more.

### Cardiovascular diseases

CVD served as the primary outcome in this study and was defined as the presence of at least one of the following self-reported conditions: heart failure, coronary heart disease, angina, heart attack, or stroke. In NHANES, information on these conditions was collected through structured interviews, based on either self-report or proxy-report. Participants were identified as having CVD if they answered “yes” to specific questions corresponding to each condition: heart failure (MCQ160b), coronary heart disease (MCQ160c), angina (MCQ160d), heart attack (MCQ160e), and stroke (MCQ160f). For analytical purposes, individuals reporting a diagnosis of any of these conditions were classified as having CVD.

### Covariates

We adjusted for a range of potential confounders that could influence physical activity levels or CVD outcomes. These included sociodemographic characteristics, lifestyle behaviors, and chronic health conditions (Supplementary material, Table S1). Sociodemographic variables consisted of age (categorized as < 65 and ≥ 65 years), sex (male or female), race/ethnicity (Mexican American, Non-Hispanic White, Non-Hispanic Black, and Other), marital status (Married/Living with Partner, Widowed/Divorced/Separated, or Never Married), educational attainment (< 9 years, 9–12 years, and > 12 years), and PIR. PIR was grouped into three categories: low income (PIR < 1), middle income (1 ≤ PIR < 4), and high income (PIR ≥ 4)^18^.

Lifestyle-related factors included body mass index (BMI), smoking status (never, former, or current), and alcohol consumption. BMI was classified into four groups according to WHO guidelines. Alcohol consumption was defined based on the Dietary Guidelines for Americans: men who consumed no more than two drinks per day and women who consumed no more than one were classified as moderate drinkers, while those who exceeded these limits were considered heavy drinkers^19^. Due to a substantial amount of missing data on alcohol use, participants with missing values were categorized separately. Chronic conditions considered in the analysis were hypertension, diabetes, and cancer.

### Statistical analysis

Survey weights were applied to account for NHANES’s complex multistage sampling design, ensuring that the findings are generalizable to the broader U.S. population. We compared participant characteristics between individuals with and without CVD. For continuous variables, results were reported as means with standard deviations (mean ± SD) and compared using independent t-tests. Categorical variables were summarized as percentages and analyzed using chi-square tests. To assess the associations between physical activity across three domains and CVD, we conducted weighted multivariable logistic regression analyses. Three models were constructed: the crude model included no covariates; Model I adjusted for sociodemographic factors; and Model II further adjusted for lifestyle variables and chronic conditions. Additionally, we conducted prespecified subgroup analyses to describe the association between physical activity and CVD risk across sociodemographic factors and performed interaction tests to formally evaluate effect modification. All statistical analyses were conducted using R software (version 4.2.2). A two-sided *P*-value of less than 0.05 was considered indicative of statistical significance.

In our primary analysis, we applied multiple imputation for all covariates except alcohol consumption. To assess the robustness of our findings, we conducted several sensitivity analyses: (1) a complete-case analysis excluding participants with any missing data; (2) an analysis excluding alcohol consumption as a covariate while adjusting for all other variables; and (3) a multiple imputation analysis that included alcohol consumption, with all missing values imputed using chained equations under the assumption of missing at random. Additionally, we conducted further sensitivity analyses to test the consistency of our results: (4) models additionally adjusted for other physical activity domains; (5) categorization of physical activity time based on tertiles instead of guideline-based thresholds; and (6) assessment of the joint associations of LTPA, TPA, and OPA. Together, these analyses allowed us to evaluate the robustness of our findings under varying model specifications, covariate adjustments, and assumptions about missing data, particularly concerning alcohol consumption.

## Results

A total of 37,879 participants were included in the analysis, with a mean age of 49.83 years (SD: 17.77), and 51.59% were female (Table 1). Compared to participants without CVD, those with CVD were more likely to be older, male, Non-Hispanic White, have a lower level of education, a higher BMI, a history of chronic conditions, and a history of smoking or current smoking status. Additionally, individuals with CVD more often reported engaging in less than 150 min of physical activity per week across all three physical activity domains.

**Table 1:** Demographic characteristics of U.S. Adults by CVD status, NHANES 2007–2020.

	Total	Without CVD	With CVD	*P*-value
N	37,879	33,718	4161	
Age (years), Mean (SD)	49.83 ± 17.77	47.81 ± 17.25	66.22 ± 12.73	< 0.001
< 65	28,712 (75.80%)	27,071 (80.29%)	1641 (39.44%)	
≥ 65	9167 (24.20%)	6647 (19.71%)	2520 (60.56%)	
Sex				< 0.001
Male	18,336 (48.41%)	16,010 (47.48%)	2326 (55.90%)	
Female	19,543 (51.59%)	17,708 (52.52%)	1835 (44.10%)	
Education level (year), n (%)				< 0.001
< 9	3884 (10.25%)	3273 (9.71%)	611 (14.68%)	
9–12	13,929 (36.77%)	12,133 (35.98%)	1796 (43.16%)	
> 12	20,066 (52.97%)	18,312 (54.31%)	1754 (42.15%)	
Marital status				< 0.001
Married/Living with Partner	22,170 (58.53%)	19,961 (59.20%)	2209 (53.09%)	
Widowed/Divorced/ Separated	8554 (22.58%)	6954 (20.62%)	1600 (38.45%)	
Never married	7155 (18.89%)	6803 (20.18%)	352 (8.46%)	
Race				< 0.001
Mexican American	5434 (14.35%)	5073 (15.05%)	361 (8.68%)	
Non-Hispanic White	15,099 (39.86%)	12,967 (38.46%)	2132 (51.24%)	
Non-Hispanic Black	8538 (22.54%)	7536 (22.35%)	1002 (24.08%)	
Other races	8808 (23.25%)	8142 (24.15%)	666 (16.01%)	
BMI (kg/m2), Mean (SD)	29.26 ± 6.89	29.12 ± 6.85	30.38 ± 7.15	< 0.001
< 18.5	569 (1.50%)	520 (1.54%)	49 (1.18%)	
18.5–24.9	9577 (25.28%)	8804 (26.11%)	773 (18.58%)	
25-29.9	13,889 (36.67%)	12,305 (36.49%)	1584 (38.07%)	
≥ 30	13,844 (36.55%)	12,089 (35.85%)	1755 (42.18%)	
Alcohol consumption, n (%)				< 0.001
Moderate drinker	10,938 (28.88%)	9744 (28.90%)	1194 (28.70%)	
Heavier drinker	11,257 (29.72%)	10,578 (31.37%)	679 (16.32%)	
Missing data	15,684 (41.41%)	13,396 (39.73%)	2288 (54.99%)	
Cancer, n (%)				< 0.001
Yes	3705 (9.78%)	2821 (8.37%)	884 (21.24%)	
No	34,174 (90.22%)	30,897 (91.63%)	3277 (78.76%)	
Diabetes, n (%)				< 0.001
Yes	6742 (17.80%)	5053 (14.99%)	1689 (40.59%)	
No	31,137 (82.20%)	28,665 (85.01%)	2472 (59.41%)	
Hypertension				< 0.001
Yes	24,036 (63.45%)	20,392 (60.48%)	3644 (87.58%)	
No	13,843 (36.55%)	13,326 (39.52%)	517 (12.42%)	
PIR				< 0.001
< 1	7388 (19.50%)	6460 (19.16%)	928 (22.30%)	
1 ≤ PIR < 4	21,891 (57.79%)	19,307 (57.26%)	2584 (62.10%)	
≥ 4	8600 (22.70%)	7951 (23.58%)	649 (15.60%)	
Smoking status				< 0.001
Never smokers	21,383 (56.45%)	19,699 (58.42%)	1684 (40.47%)	
Ever smokers	8929 (23.57%)	7324 (21.72%)	1605 (38.57%)	
Current smokers	7567 (19.98%)	6695 (19.86%)	872 (20.96%)	
TPA				< 0.001
< 150 min/week	32,717 (86.37%)	28,888 (85.68%)	3829 (92.02%)	
≥ 150 min/week	5162 (13.63%)	4830 (14.32%)	332 (7.98%)	
OPA				< 0.001
< 150 min/week	25,019 (66.05%)	21,906 (64.97%)	3113 (74.81%)	
≥ 150 min/week	12,860 (33.95%)	11,812 (35.03%)	1048 (25.19%)	
LTPA				< 0.001
< 150 min/week	25,590 (67.56%)	22,223 (65.91%)	3367 (80.92%)	
≥ 150 min/week	12,289 (32.44%)	11,495 (34.09%)	794 (19.08%)	

CVD, Cardiovascular disease; LTPA, Leisure-time physical activity; OPA, Occupational physical activity; TPA, Transportation-related physical activity; PIR, Poverty-income ratio; BMI, body mass index.

The associations between physical activity domains and CVD were examined using weighted logistic regression (Table 2). In the crude model, participants who met the recommended ≥ 150 min of weekly physical activity had significantly lower odds of CVD compared to those who engaged in less than 150 min. These associations remained significant for LTPA and TPA after adjusting for sociodemographic factors (Model I) and further adjusting for lifestyle behaviors and chronic health conditions (Model II). However, OPA of ≥ 150 min per week was not significantly associated with reduced CVD risk in the fully adjusted model. Specifically, participants reporting ≥ 150 min of LTPA had a 22% lower likelihood of CVD [OR: 0.78 (95% CI: 0.70–0.88)], and those reporting ≥ 150 min of TPA had a 40% lower likelihood [OR: 0.60 (95% CI: 0.50–0.72)].

To evaluate the robustness of our findings, we conducted a series of sensitivity analyses. First, results from the complete-case analysis and the model excluding alcohol consumption as a covariate were consistent with those of the primary analysis (Supplementary Tables S2–S3). We further included alcohol consumption in the imputation process and found similar associations (Supplementary Table S4). Additional sensitivity analyses also confirmed the robustness of our results: adjusting for other physical activity domains (Supplementary Table S5), categorizing physical activity time using tertiles rather than guideline-based thresholds (Supplementary Table S6), and examining the joint associations of LTPA, OPA, and TPA (Supplementary Table S7). In the joint analysis, compared with the reference group (low LTPA and low OPA), only the group with high LTPA and low OPA had a significantly lower risk of CVD (OR: 0.81, 95% CI: 0.70–0.94). High OPA alone or in combination with high LTPA was not associated with a statistically significant reduction in CVD risk. A similar pattern was observed in the joint analysis of TPA and OPA. These findings are consistent with the primary results and further support the robustness of our conclusions across different model specifications and assumptions.

**Table 2:** Association between physical activity domains and CVD among U.S. adults, NHANES 2007–2020, weighted.

	Crude model, *P*-value	Model I, *P*-value	Model II, *P*-value
LTPA			
< 150 min/week	Reference	Reference	Reference
≥ 150 min/week	0.47 (0.42; 0.53), < 0.01	0.63 (0.57; 0.71), < 0.01	0.78 (0.70; 0.88), < 0.01
TPA			
< 150 min/week	Reference	Reference	Reference
≥ 150 min/week	0.46 (0.39; 0.55), < 0.01	0.53 (0.44; 0.64), < 0.01	0.60 (0.50; 0.72), < 0.01
OPA			
< 150 min/week	Reference	Reference	Reference
≥ 150 min/week	0.67 (0.61; 0.74), < 0.01	0.93 (0.82; 1.05), 0.16	0.94 (0.84; 1.06), 0.22

Crude model, unadjusted; Model I, adjusted for sociodemographic characteristics (age, gender, race, marital status, PIR, and education levels); Model II, further adjusted for lifestyle behaviors (BMI, smoke, and alcohol consumption) and chronic health conditions (hypertension, cancer, and diabetes). Abbreviations: LTPA, Leisure-time physical activity; OPA, Occupational physical activity; TPA, Transportation-related physical activity.

**Fig. 2:**
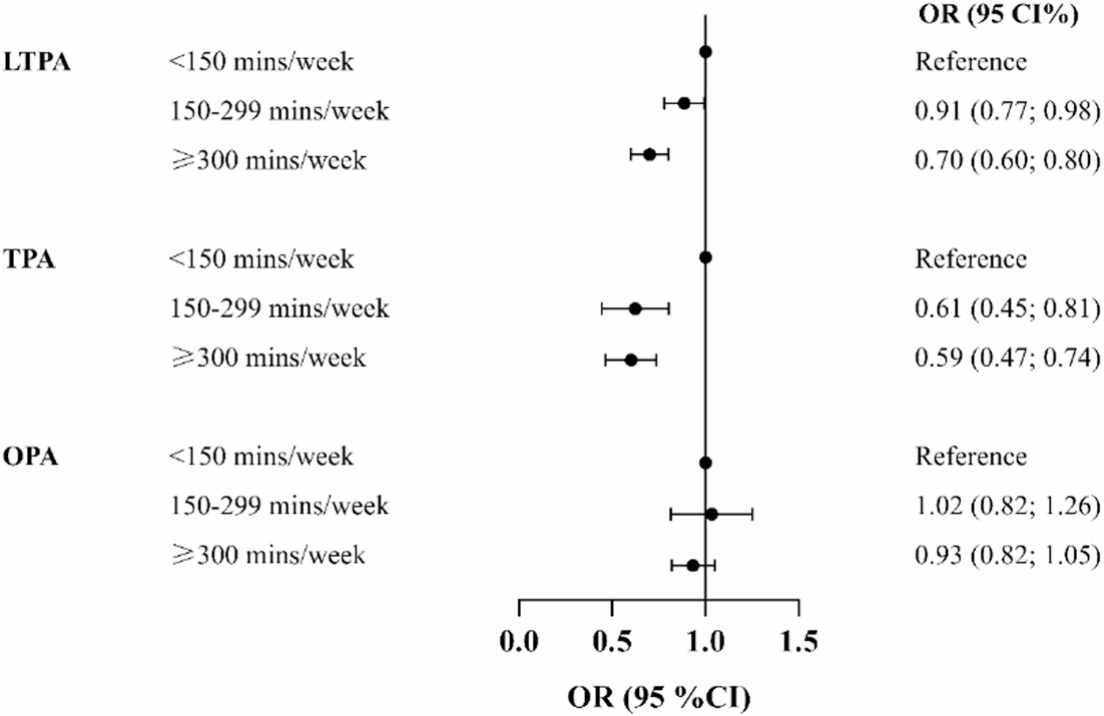
Association between physical activity domains and CVD by activity level among U.S. adults, NHANES 2007–2020, Weighted. Abbreviations: CVD, Cardiovascular disease; LTPA, Leisure-time physical activity; OPA, Occupational physical activity; TPA, Transportation-related physical activity.

To explore potential dose-response relationships, weekly minutes of physical activity were categorized into three groups: <150 min, 150–299 min, and ≥ 300 min (Fig. 2). After full adjustment for covariates, compared to those reporting < 150 min of weekly LTPA, participants engaging in 150–299 min had a 9% lower odds of CVD [OR: 0.91 (95% CI: 0.77–0.98)], and those with ≥ 300 min had a 30% lower odds [OR: 0.70 (95% CI: 0.60–0.80)]. A similar pattern was observed for TPA: participants with 150–299 min and ≥ 300 min per week had 39% [OR: 0.61 (95% CI: 0.45–0.81)] and 41% [OR: 0.59 (95% CI: 0.47–0.74)] lower odds of CVD, respectively, compared to those with < 150 min. In contrast, OPA was not significantly associated with lower CVD risk in any duration category.

**Fig. 3:**
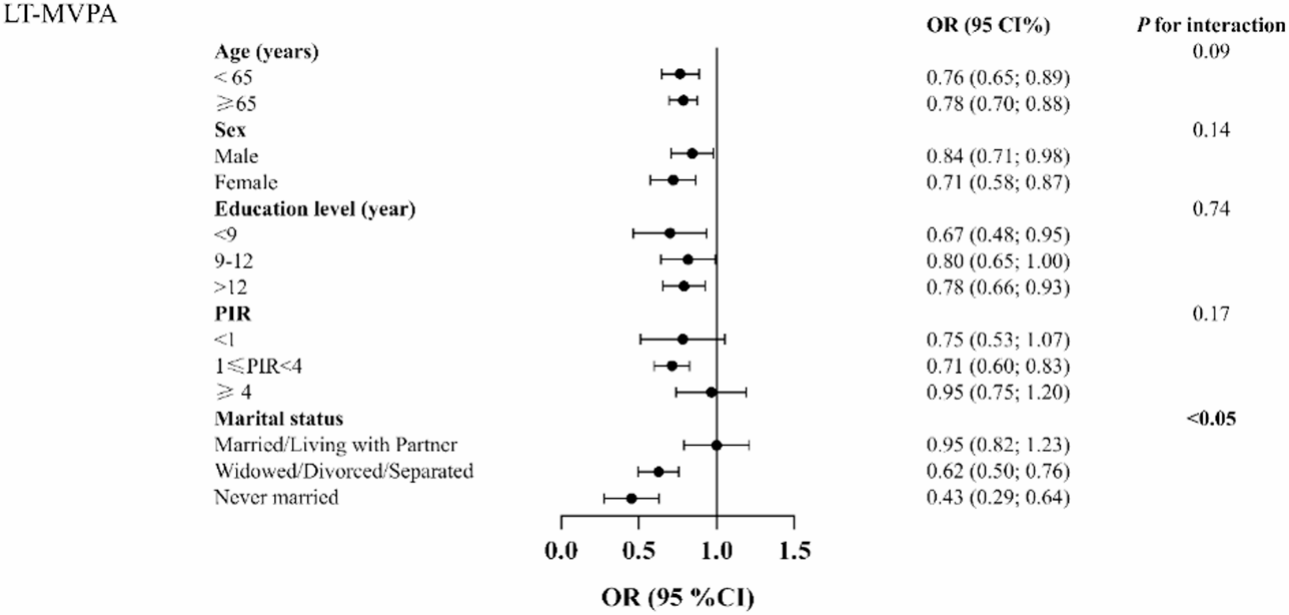
Subgroup analyses and interaction tests for the association between LTPA and CVD, among U.S. adults, NHANES 2007–2020, weighted. Abbreviations: PIR, Poverty-income ratio.

**Fig. 4:**
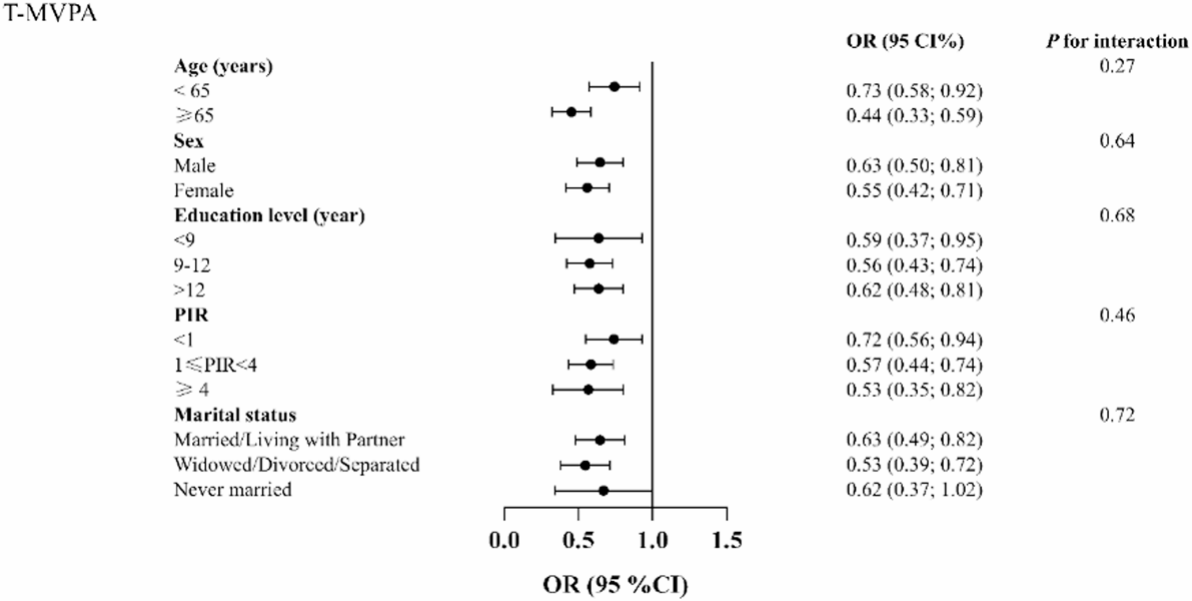
Subgroup analyses and interaction tests for the association between TPA and CVD, among U.S. adults, NHANES 2007–2020, weighted. Abbreviations: PIR, Poverty-income ratio.

Figure 3 presents subgroup analyses and interaction tests for the association between LTPA and CVD by age, sex, PIR, marital status, and education. An inverse association between ≥ 150 min of LTPA and CVD was observed in most subgroups, although it was not statistically significant among participants who were married or living with a partner, and among those with PIR < 1 or PIR ≥ 4. A statistically significant interaction was observed only for marital status, indicating that the association between LTPA and CVD differed by marital status, whereas no significant effect modification was found for the other sociodemographic factors. Similarly, Fig. 4 shows subgroup analyses for TPA, where the interaction tests were not significant. The significantly inverse association between ≥ 150 min of TPA and CVD was consistent across all subgroups except those who had never been married.

## Discussion

In this nationally representative sample of U.S. adults, we found that meeting the minimum recommended level of physical activity (≥ 150 min per week) through LTPA or TPA was significantly associated with lower odds of CVD, compared to individuals who did not meet the guidelines. Moreover, a clear dose-response relationship was observed: those engaging in ≥ 300 min per week, double the current recommendation, experienced greater reductions in CVD risk. These findings suggest that not only meeting but exceeding physical activity recommendations may confer additional cardiovascular benefits. In contrast, OPA showed no significant association with CVD risk across different duration categories, reinforcing the idea that the health effects of physical activity vary by context.

Our findings are consistent with a growing body of evidence suggesting that the health benefits of physical activity are domain-specific. LTPA, which is typically voluntary, structured, and includes adequate rest, has been consistently linked to a wide range of positive health outcomes. For example, a prospective study from Finland found that individuals engaging in high levels of vigorous LTPA (> 3 h per week) had a reduced risk of all stroke subtypes compared to those who were inactive^20^. This finding is supported by another study showing that higher LTPA levels are inversely associated with the risk of transient ischemic attack^21^. A meta-analysis further reported that greater participation in LTPA is associated with a 23% lower risk of stroke, a 27% lower risk of heart failure, and an 18% lower risk of myocardial infarction^22^.

The benefits of LTPA extend beyond cardiovascular health. A large cohort study found that higher levels of LTPA were associated with reduced risks for 13 different types of cancer, including a 42% lower risk of esophageal adenocarcinoma and a 10% lower risk of breast cancer^23^. In mental health, a cross-sectional study from China reported that individuals with higher LTPA levels had a 21% lower likelihood of experiencing depression^24^. Likewise, a U.S.-based cross-sectional study found that greater engagement in LTPA was associated with healthier blood cholesterol profiles^25^. Additionally, another study reported that individuals who engaged in moderate levels of vigorous LTPA (more than 4 h of physical activity per week) had a 33% lower risk of developing diabetes, while those with high levels of vigorous LTPA (> 3 h per week) had a 39% lower risk, compared to inactive individuals^26^.

Similarly, TPA, often achieved through walking or cycling for commuting, has been shown to offer health benefits, likely due to its moderate intensity and regular nature. A study using NHANES 2007–2010 data examined the association between TPA and health outcomes, finding that compared to individuals without TPA, those engaging in low (1–149 min) and high (≥ 150 min) levels of TPA had lower BMI and waist circumference^27^. Additionally, high TPA (≥ 150 min) was associated with a 31% reduced risk of hypertension and diabetes^27^. Another study also found that higher levels of TPA were linked to an 18% lower odds of myocardial infarction^22^. In line with these findings, our study identified a dose-response relationship between TPA and CVD, with greater weekly engagement in TPA corresponding to progressively lower odds of CVD.

Conversely, no significant cardiovascular benefits were observed for OPA, even at higher levels of activity. This finding aligns with the so-called “physical activity paradox”, which suggests that physical exertion performed as part of one’s occupation may not confer the same health benefits as leisure-time activity and may even be detrimental^28^. OPA often involves repetitive tasks, prolonged standing, physical strain, and limited recovery time, all of which can contribute to physiological stress rather than protection^29^. For instance, a study among Danish workers found that men in the highest quartile of OPA had a 79% increased risk of all-cause mortality compared to those in the lowest quartile^30^. Another study, also conducted in Denmark, objectively assessed OPA and LTPA in blue-collar workers and used heart rate monitors to evaluate heart rate and heart rate variability during sleep as markers of autonomic regulation. The results showed that both higher OPA and LTPA were inversely associated with autonomic regulation during sleep, and that the beneficial effects of LTPA were diminished in workers with high levels of OPA, suggesting that occupational strain may counteract the positive impact of recreational physical activity^31,32^. Consistent with this, our joint analysis revealed that only individuals with high LTPA and low OPA experienced a significantly lower risk of CVD, while high OPA alone or in combination with high LTPA was not associated with a significant risk reduction. A similar pattern emerged in the joint analysis of TPA and OPA, where cardiovascular benefits were observed only among those with high TPA and low OPA. These findings reinforce the hypothesis that OPA may not only lack cardiovascular benefit but may also attenuate the protective effects of other forms of physical activity.

Another interesting finding is that the association between LTPA and CVD varied significantly by marital status. Specifically, meeting the recommended ≥ 150 min per week of LTPA was significantly associated with a lower risk of CVD among individuals who were widowed, divorced, separated, or never married, but not among those who were married or partnered. One possible explanation is that married or partnered individuals may already experience a lower baseline CVD risk due to protective factors such as emotional support and healthier lifestyles^33^, which could attenuate the additional benefits of LTPA in this group. In contrast, individuals who are unmarried, separated, or widowed may lack these protective social and behavioral factors^33^, making physical activity a more prominent and independent contributor to cardiovascular health. Additionally, studies have shown that couples tend to exhibit similar cardiovascular risk profiles, especially in diet and exercise habits, making it harder to isolate the specific impact of LTPA among married or partnered individuals^34^. Relationship quality may also play a role, those in strained partnerships may experience stress-related CVD risk, potentially offsetting the benefits of LTPA^35^.

Moreover, in this study, CVD encompasses five diseases (angina, heart attack, stroke, congestive heart failure, and chronic heart disease), which, while having distinct pathophysiologies, share common risk factors, such as hypertension, diabetes, and dyslipidemia, that are closely linked to physical inactivity, as well as similar clinical outcomes, including increased morbidity and mortality^36^. Given these shared characteristics, we grouped them under a single CVD category to increase statistical power and provide a broader understanding of the relationship between physical activity and CVD risk. While this approach enhances power, it may obscure important differences between the pathologies, potentially leading to an oversimplification of the associations observed. Therefore, caution is needed when interpreting our findings, as the results may not fully reflect the nuances of each individual pathology within the CVD spectrum.

Our study offers several strengths, including the use of a large, diverse, and nationally representative sample, detailed assessment of physical activity across domains, and extensive adjustment for potential confounding factors. By examining LTPA, OPA, and TPA separately, we provide more nuanced insights into how physical activity context may shape its health effects, information that could help refine public health recommendations.

However, several limitations should be acknowledged. First, the cross-sectional design limits causal inference, and reverse causality cannot be ruled out. Individuals with pre-existing CVD may reduce their overall physical activity, including both LTPA and TPA, due to health-related limitations or concerns about exacerbating their condition. However, OPA may be less affected, as it is primarily influenced by the physical demands of their occupation, which may not be easily adjusted based on personal choice. It is also possible that higher levels of OPA could initially be associated with higher CVD risk. Yet, due to reverse causality, individuals with CVD might eventually reduce their OPA as their condition progresses, which could explain the lack of a significant relationship observed in our study.

Second, all physical activity measures and CVD diagnoses were self-reported, which may introduce recall or misclassification bias. These biases may differentially affect the three domains: OPA is likely more accurately reported due to its structured nature^37^, while LTPA may be overestimated due to recall or social desirability bias^38^ and TPA may vary depending on daily routine and reporting accuracy^39^. Third, we did not capture the intensity, context, or continuity of physical activity, nor did we assess occupational stress, all of which may further influence the observed associations. Lastly, although we adjusted for many potential confounders, residual confounding from unmeasured factors cannot be ruled out.

## Conclusion

This study shows that meeting or exceeding the recommended levels of LTPA and TPA is associated with a lower risk of CVD among U.S. adults, with even greater benefits observed at higher levels of activity. Promoting active transportation (e.g., walking or cycling) and encouraging regular participation in LTPA could be effective strategies to reduce CVD risk. In contrast, no significant association was found for OPA, which may not confer cardiovascular benefits due to its repetitive, static, or time-pressured nature. To mitigate CVD risk in high-OPA workers, incorporating regular breaks, task variation, and ergonomic support should be considered.

## Supplementary Information

Below is the link to the electronic supplementary material.

Supplementary Material 1

## Data Availability

The data used in this study are publicly available from the National Health and Nutrition Examination Survey (NHANES) at [https://www.cdc.gov/nchs/nhanes/index.html](https:/www.cdc.gov/nchs/nhanes/index.html) (accessed on May 1, 2025).
