# Supplementary material for: Association between physical activity domains and cardiovascular diseases among US adults: evidence from NHANES 2007–2020
Source: Sci Rep. 2025 Oct 9;15:35341. doi: 10.1038/s41598-025-19419-3 (PMC12511566; doi:10.1038/s41598-025-19419-3)
Supplement: Supplementary file 1 — Supplementary Material 1 [file 41598_2025_19419_MOESM1_ESM.docx]

**Supplementary material**

**Table S1.** Covariates included in multivariable logistic regression models in the primary analysis.

|  | **Variable Name** | **Type** | **Coding / Description** | **Source / Section** |
| --- | --- | --- | --- | --- |
| Sociodemographic characteristics | Age | Categorical | < 65 years / ≥65 years | Demographics |
|  | Sex | Categorical | Male /female | Demographics |
|  | Education level | Categorical | <9 years, 9-12 years, >12 years | Demographics |
|  | Race | Categorical | Mexican American, Non-Hispanic White, Non-Hispanic Black, Other races | Demographics |
|  | Marital status | Categorical | Married/Living with Partner,  Widowed/Divorced/Separated,  Never married | Demographics |
|  | PIR | Categorical | Low income (PIR < 1), middle income (1 ≤ PIR < 4), and high income (PIR ≥ 4) | Demographics |
| Lifestyle behaviors | Smoking | Categorical | Never smokers, ever smokers,  current smokers | Questionnaire (Smoking - Cigarette Use – SMQ) |
|  | Alcohol consumption | Categorical | Moderate drinker, Heavier drinker, Missing data | Questionnaire (Alcohol use – ALQ) |
|  | BMI | Categorical | <18.5 kg/m2, 18.5-24.9 kg/m2, 25-29.9 kg/m2, ≥30 kg/m2 | Examination Data (Body Measures) |
| Chronic health conditions | Hypertension | Categorical | Yes / No | Examination Data (Blood Pressure) and Questionnaire (Blood Pressure & Cholesterol) |
|  | Cancer | Categorical | Yes / No | Questionnaire (Medical Conditions) |
|  | Diabetes | Categorical | Yes / No | Laboratory Data (Glycohemoglobin, Plasma Fasting Glucose) and Questionnaire (Diabetes) |

**Abbreviations:** PIR, Poverty-income ratio; BMI, body mass index.

**Table S2.** Sensitivity analysis of association between physical activity domains and CVD among U.S. adults (NHANES 2007-2020), excluding alcohol consumption.

|  | **Full adjusted model** | ***P*-value** |
| --- | --- | --- |
| **LTPA** |  |  |
| <150 mins/week | Reference |  |
| ≥150 mins/week | 0.76 (0.68; 0.85) | 0.16 |
| **TPA** |  |  |
| <150 mins/week | Reference |  |
| ≥150 mins/week | 0.59 (0.49; 0.71) | <0.01 |
| **OPA** |  |  |
| <150 mins/week | Reference |  |
| ≥150 mins/week | 0.92 (0.82; 1.03) | <0.01 |

Full adjusted model: adjusted for sociodemographic characteristics (age, gender, race, marital status, PIR, and education levels); lifestyle behaviors (BMI and smoke) and chronic health conditions (hypertension, cancer, and diabetes). **Abbreviations:** LTPA, Leisure-time physical activity; OPA, Occupational physical activity; TPA, Transportation-related physical activity; PIR, Poverty-income ratio; BMI, body mass index.

**Table S3.** Sensitivity analysis of association between physical activity domains and CVD among U.S. adults (NHANES 2007-2020), complete case analysis (N = 17490).

|  | **Full adjusted model** | ***P*-value** |
| --- | --- | --- |
| **LTPA** |  |  |
| <150 mins/week | Reference |  |
| ≥150 mins/week | 0.77 (0.67; 0.90) | <0.01 |
| **TPA** |  |  |
| <150 mins/week | Reference |  |
| ≥150 mins/week | 0.61 (0.46; 0.80) | <0.01 |
| **OPA** |  |  |
| <150 mins/week | Reference |  |
| ≥150 mins/week | 0.95 (0.82; 1.09) | 0.46 |

Full adjusted model: adjusted for sociodemographic characteristics (age, gender, race, marital status, PIR, and education levels); lifestyle behaviors (BMI, smoke, and alcohol consumption) and chronic health conditions (hypertension, cancer, and diabetes). **Abbreviations:** LTPA, Leisure-time physical activity; OPA, Occupational physical activity; TPA, Transportation-related physical activity; PIR, Poverty-income ratio; BMI, body mass index.

**Table S4.** Sensitivity analysis of association between physical activity domains and CVD among U.S. adults (NHANES 2007-2020), multiple imputation for all covariates.

|  | **Full adjusted model** | ***P*-value** |
| --- | --- | --- |
| **LTPA** |  |  |
| <150 mins/week | Reference |  |
| ≥150 mins/week | 0.75 (0.67; 0.84) | < 0.01 |
| **TPA** |  |  |
| <150 mins/week | Reference |  |
| ≥150 mins/week | 0.58 (0.49; 0.70) | < 0.01 |
| **OPA** |  |  |
| <150 mins/week | Reference |  |
| ≥150 mins/week | 0.92 (0.82; 1.03) | 0.16 |

Full adjusted model: adjusted for sociodemographic characteristics (age, gender, race, marital status, PIR, and education levels); lifestyle behaviors (BMI, smoke, and alcohol consumption) and chronic health conditions (hypertension, cancer, and diabetes). **Abbreviations:** LTPA, Leisure-time physical activity; OPA, Occupational physical activity; TPA, Transportation-related physical activity; PIR, Poverty-income ratio; BMI, body mass index.

**Table S5.** Sensitivity analysis of associations between physical activity domains and CVD among U.S. adults (NHANES 2007-2020), mutually adjusted for other activity domains.

|  | **Full adjusted model** | ***P*-value** |
| --- | --- | --- |
| **LTPA ^a^** |  |  |
| <150 mins/week | Reference |  |
| ≥150 mins/week | 0.81 (0.72; 0.91) | <0.01 |
| **TPA ^b^** |  |  |
| <150 mins/week | Reference |  |
| ≥150 mins/week | 0.64 (0.53; 0.77) | <0.01 |
| **OPA ^c^** |  |  |
| <150 mins/week | Reference |  |
| ≥150 mins/week | 0.95 (0.85; 1.07) | 0.43 |

Full adjusted model: adjusted for sociodemographic characteristics (age, gender, race, marital status, PIR, and education levels); lifestyle behaviors (BMI, smoke, and alcohol consumption) and chronic health conditions (hypertension, cancer, and diabetes). ^a^ Additionally adjusted for TPA and OPA; ^b^ Additionally adjusted for LTPA and OPA; ^c^ Additionally adjusted for LTPA and TPA. **Abbreviations:** LTPA, Leisure-time physical activity; OPA, Occupational physical activity; TPA, Transportation-related physical activity; PIR, Poverty-income ratio; BMI, body mass index.

**Table S6.** Sensitivity analysis of associations between physical activity domains (tertile categories) and CVD among U.S. adults (NHANES 2007-2020).

|  | **Full adjusted model** | ***P*-value** |
| --- | --- | --- |
| **LTPA** |  |  |
| Tertile 1 | Reference |  |
| Tertile 2 | 0.64 (0.56; 0.72) | <0.01 |
| Tertile 3 | 0.65 (0.57; 0.75) | <0.01 |
| **TPA** |  |  |
| Tertile 1 | Reference |  |
| Tertile 2 | 0.64 (0.56; 0.72) | <0.01 |
| Tertile 3 | 0.65 (0.57; 0.75) | <0.01 |
| **OPA** |  |  |
| Tertile 1 | Reference |  |
| Tertile 2 | 0.99 (0.89; 1.13) | 0.98 |
| Tertile 3 | 0.96 (0.85; 1.09) | 0.54 |

Full adjusted model: adjusted for sociodemographic characteristics (age, gender, race, marital status, PIR, and education levels); lifestyle behaviors (BMI, smoke, and alcohol consumption) and chronic health conditions (hypertension, cancer, and diabetes). **Abbreviations:** LTPA, Leisure-time physical activity; OPA, Occupational physical activity; TPA, Transportation-related physical activity; PIR, Poverty-income ratio; BMI, body mass index.

**Table S7.** Joint associations of LTPA, TPA, and OPA with CVD among U.S. Adults (NHANES 2007-2020).

|  | **N (%)** | **Full adjusted model** | ***P*-value** |
| --- | --- | --- | --- |
| **LTPA** |  |  |  |
| Low LTPA & low OPA | 17845 (47.11) | Reference |  |
| Low LTPA & high OPA | 7745 (20.45) | 0.87 (0.75; 1.02) | 0.07 |
| High LTPA & low OPA | 7174 (18.94) | 0.81 (0.70; 0.94) | **<0.01** |
| High LTPA & high OPA | 5115 (13.55) | 0.91 (0.77; 1.08) | 0.29 |
| **TPA** |  |  |  |
| Low TPA & low OPA | 22002 (58.05) | Reference |  |
| Low TPA & high OPA | 10715 (28.29) | 1.02 (0.90; 1.15) | 0.75 |
| High TPA & low OPA | 3017 (7.96) | 0.64 (0.51; 0.79) | **<0.01** |
| High TPA & high OPA | 2145 (5.66) | 0.82 (0.61; 1.09) | 0.18 |

Full adjusted model: adjusted for sociodemographic characteristics (age, gender, race, marital status, PIR, and education levels); lifestyle behaviors (BMI, smoke, and alcohol consumption) and chronic health conditions (hypertension, cancer, and diabetes). **Abbreviations:** LTPA, Leisure-time physical activity; OPA, Occupational physical activity; PIR, Poverty-income ratio; BMI, body mass index.
